# Construction of a clinically significant prostate cancer risk prediction model based on traditional diagnostic methods

**DOI:** 10.3389/fonc.2024.1474891

**Published:** 2024-12-20

**Authors:** Wen-Tong Ji, Yong-Kun Wang, Zhan-Yang Han, Si-Qi Wang, Yao Wang

**Affiliations:** ^1^ Urology 2nd Department, China-Japan Union Hospital of Jilin University, Changchun, Jilin, China; ^2^ Orthopedics Department, China-Japan Union Hospital of Jilin University, Changchun, Jilin, China; ^3^ Urology Department, Shuangyang People’s Hospital, Changchun, Jilin, China; ^4^ Department of Ophthalmology, China-Japan Union Hospital of Jilin University, Changchun, Jilin, China; ^5^ Jilin Key Laboratory of Molecular Diagnosis of Urologic Neoplasms, Urology 2nd Department, China-Japan Union Hospital of Jilin University, Changchun, Jilin, China

**Keywords:** prostate biopsy, clinically significant prostate cancer, risk prediction model, diagnosis, nomogram

## Abstract

**Objectives:**

to construct a prediction model for clinically significant prostate cancer (csPCa) based on prostate-specific antigen (PSA) levels, digital rectal examination (DRE), and transrectal ultrasonography (TRUS).

**Methods:**

We retrospectively analysed 1196 Asian patients who underwent transrectal ultrasound-guided biopsy (TRUSB) between June 2000 and February 2023. Patients were randomly divided into a training set of 837 cases (70%) and a validation set of 359 patients (30%). A csPCa risk prediction model was established using the logistic regression. The performance of the model was examined based on calibration curves, receiver operating characteristic (ROC) curves, decision curve analysis (DCA), and clinical impact curves (CIC).

**Results:**

Serum PSA levels, age, DRE results, prostatic shape, prostatic border and hypoechoic area were associated with pathological outcomes. The area under the ROC curve of the training set was 0.890 (95%CI: 0.865-0.816). The optimal cut-off value was 0.279. The calibration curves indicated good calibration, and the DCA and CIC results demonstrated good clinical utility. Significantly, the prediction model has higher negative predictive value (89.8%) and positive predictive value (68.0%) compared with MRI. Subsequently, we developed an online calculator (https://jiwentong0.shinyapps.io/dynnomapp/) with six variables for biopsy optimization.

**Conclusion:**

This study incorporated the results of three traditional diagnostic methods to establish a cost-effective and highly accurate model for predicting csPCa before biopsy. With this model, we aim to provide a non-invasive and cost-effective tool for csPCa detection in Asia and other underdeveloped areas.

## 1 Introduction

Prostate cancer (PCa) poses a significant threat to human health. According to 2020 statistical data, prostate cancer ranks second in terms of cancer incidence and fifth in terms of cancer mortality among males (1). Thus, PCa diagnosis is vital in guiding treatment and reducing the suffering and mortality of patients with PCa (2).

According to the European Association of Urology (EAU) Guidelines, a biopsy is recommended if there is a suspicion of PCa based on abnormality on digital rectal examination (DRE) or an elevated level of serum prostate-specific antigen (PSA) (3). However, elevated PSA level is not specific to PCa and may be observed in other conditions such as benign prostatic hyperplasia (BPH) and prostatitis (4). While a serum total PSA level of 4.0 ng/mL is recommended as a cut-off value, 25% of men with PCa may have a PSA below 4.0 ng/mL (5). For DRE, the possible signs of PCa include induration and nodularity; however, this examination is subjective, and the results may show considerable interindividual variation. Consequently, only 50% of men with suspicious DRE findings actually have PCa (6).

Therefore, to reduce overdiagnosis and potential complications caused by unnecessary biopsies (7), the EAU, as well as the American Urological Association, support the use of prostate MRI for biopsy optimization (8, 9). However, the routine use of prostate MRI prior to the initial biopsy should still be carefully considered. On the one hand, the variable diagnostic accuracy of clinically significant PCa (csPCa) (10) and relatively low negative predictive value (NPV)(85%) and positive predictive value (PPV) (27%−44%) (11) remain challenges in the clinical application of this approach. On the other hand, using a prostate MRI would require upfront costs (12). In some developing countries of Asia, Africa, and Latin America, it is not uncommon that patients with elevated PSA levels only and without any other discomfort would reject prostate MRI for financial reasons. Given this requirement, we realized the necessity of a cost-effective and accurate tool to provide diagnostic information for those who cannot afford the use of an MRI before an initial biopsy.

Compared with prostate MRI, transrectal ultrasound (TRUS) is a cheaper and more easily accessible imaging tool used for prostate evaluation (13). Grayscale TRUS imaging of the prostate is the basic method for the diagnostic evaluation of PCa (14). Although PCa is commonly asymptomatic at the early stage, many cancer foci can still be detected using TRUS. Hypoechoic nodules on grayscale TRUS can be used to predict PCa (15).

Therefore, in this study, we aimed to design a cost-effective, non-invasive, and highly accurate csPCa risk prediction model to provide an auxiliary diagnosis before initial biopsy, investigate the combined effect of these three traditional diagnostic methods (PSA testing, TRUS, and DRE), and explore the approaches to improve their diagnostic value.

## 2 Materials and methods

### 2.1 Data source

This was a single-centre, retrospective study. The study followed the guidelines for transparent reporting of a multivariable prediction model for individual prognosis or diagnosis (TRIPOD) (16). The clinical data of all patients who underwent TRUS-guided biopsy at this centre between June 2000 and February 2023 were consecutively recorded. Data of 2477 patients were obtained for research purposes from 01/01/2024 to 01/02/2024.

### 2.2 Inclusion and exclusion criteria

The inclusion criteria were as follows: (i) patients who underwent TRUSB between June 2000 and February 2023; (ii) patients who underwent PSA, TRUS and DRE before initial biopsy; and (iii) the patient had a definitive pathological diagnosis. Exclusion criteria were as follows: (i) PSA> 100ng/mL (ii) > 30% missing data (after meeting the inclusion criteria); (iii) patients whose pathological reports were not available or the diagnosis was unknown; (iv) the patient could not tolerate or did not complete the biopsy.

Among the 2477 patients, two patients’ pathological reports had indecipherable handwriting, one patient did not cooperate causing the biopsy to fail, and 971 subjects had no pathological diagnosis or their results were not recorded at that time. A total of 1503 subjects were included in the study according to the inclusion criteria. A total of 279 subjects among the 1503 patients were excluded because of missing data, and 28 patients were excluded for PSA higher than 100ng/mL. Finally, 1196 patients were included in this study.

### 2.3 Parameters selection and collection

A total of 9 candidate variables were selected, including i) age, ii) serum PSA, iii) result of DRE, iv) prostate volume, v) prostatic border, vi) shape, vii) hypoechoic area, viii) condition of seminal vesicle, ix) csPCa diagnosis. Among them, i, ii, and iv are continuous variables, iii, v, vi, vii, viii are binary variables, and ix is the dependent variable. csPCa was defined according to the EAU guidelines: International Society for Urological Pathology (ISUP) 2 or higher (3) or the Epstein criteria: Gleason score (GS) > 6 or GS 6 with ≥ 50% of cancer per core involvement or > 2 cores with cancer (17).

Blood samples were drawn before ultrasonography and biopsy and before or at least one week after rectal examination, which may have increased the serum PSA concentration. Considering DRE results are highly subjective, we categorised apparent induration and nodularity as abnormal. Prostate volume, prostatic border, shape, hypoechoic area, and the condition of seminal vesicle were obtained from grey-scale TRUS. The prolate ellipsoid formula estimated the volume of the prostate: (18)


length (L) x height (H) x width (W) x π/6 (0.52)

We defined the abnormal and normal prostatic border, shape, hypoechoic area, and the condition of the seminal vesicle according to whether the border is clear, whether the shape is symmetrical, whether the prostate contains a hypoechoic area, and whether the seminal vesicle has uneven echoes and (or) indistinct border respectively. The abnormal examples of prostatic border, shape, hypoechoic area, and the condition of the seminal vesicle are shown in **Supplementary Material 1**. To reduce errors in the process of TRUS and DRE, every patient will undergo the first examination after being admitted to the hospital and the second before TRUS-guided biopsy (TRUSB). Verification during a third examination resolved any significant differences between the two data sets. The prostate biopsy was uniformly performed using a transperineal approach, with 12 cores.

### 2.4 Statistical analysis

Statistical analyses were performed using R software V.4.3.3 (R Core Team, Vienna, Austria, available at https://www.R-project.org). Multiple imputations were applied to fill in missing data (using “mice” package, m=4, method=cart, seed=1024). The Mann-Whitney U test was used for continuous data, and the chi-square test was used for categorical data to verify there were no significant differences in the dataset before and after multiple imputations. The 1196 individuals were randomly divided into two sets: a training set including 837 males and a validation set including 359 males. Univariate logistic regression analysis was performed on the training set to screen for potential factors influencing the dependent variable. Variables with a *p*-value >0.05 were excluded. Multivariate analysis and forward, backward, and forward-backward stepwise logistic regression were used to generate four novel predictive models. We chose the model with the lowest Akaike information criterion (AIC) value. A nomogram was used to visualize the mathematical model. The area under the curve (AUC), goodness-of-fit test, decision curve analysis (DCA), and clinical impact curve (CIC) were used to evaluate the performance and clinical utility of the model.

In addition, in order to prove that our model developed with the combined data from PSA, TRUS, and DRE had better performance than these three screening methods being used separately, we developed three prediction models with PSA (serum PSA), TRUS (prostatic border, shape, hypoechoic area) and DRE data only.

We further developed an online interface written in R using the Shiny framework (http://www.shinyapps.io/) as a user-friendly tool.

## 3 Results

### 3.1 Patient characteristics

The results Mann-Whitney U test and chi-square test revealed no significant differences in the dataset before and after multiple imputations (**Supplementary Material 2** **).** A total of 1196 individuals were included in the analysis. Based on the pathological diagnosis, the incidence of csPCa in patients undergoing TRUSB was 30.4% (364/1196). **Table 1** lists the baseline characteristics of patients in the training and validation sets.

**Table 1:** Basic demographic and clinical characteristics of the training and validation sets.

Variables	Total (n=1196)	Training set (n=837)	Validation set (n=359)	*p* value
DRE, n, (%)				0.87
Normal	819, (68.48)	572, (68.34)	247, (68.80)	
Abnormal	377, (31.52)	265, (31.66)	112, (31.20)	
Border, n, (%)				0.31
Clear	762, (63.71)	541, (64.64)	211, (61.56)	
Blurred	434, (36.29)	296, (35.36)	138, (38.44)	
Shape, n, (%)				0.92
Symmetrical	894, (74.75)	625, (74.67)	269, (74.93)	
Asymmetrical	302, (25.25)	212, (25.33)	90, (25.07)	
Hypoechoic area, n, (%)				0.71
Not found	650, (54.35)	452, (54.00)	198, (55.15)	
Found	546, (45.65)	388, (46.00)	161, (44.85)	
Seminal vesicle, n, (%)				0.64
Normal	1054, (88.13)	740, (88.41)	314, (87.47)	
Abnormal	142, (11.87)	97, (11.59)	45, (12.53)	
Age (IQR, year)	71, (64.76)	71, (65,76)	70, (63,76)	0.17
PSA (IQR, ng/ml)	17.10, (8.80, 39.70)	17.50, (9.00,40.90)	16.00, (8.30, 35.00)	0.31
Prostate volume (IQR, cm^3^)	55.90, (36.60,88.00)	56.00, (35.80,88.00)	55.35, (37.70,87.70)	0.26

DRE, digital rectal examination; PSA, prostate-specific antigen; IQR, interquartile range.

p>0.05 indicates that there is no statistically significant difference of data between training set and validation set.

### 3.2 Selection of predictors and construction of nomogram model

Univariate logistic regression analysis was performed preliminarily for the independent variables. The following seven factors were significantly associated with csPCa in the univariate analysis: serum PSA level, DRE results, age, prostatic border, shape, hypoechoic area, and seminal vesicle condition (*p* < 0.05). Their association with pathological outcome were further tested by multivariate analysis, in which “prostatic border” was removed. Finally, six independent variables were applied to construct the prediction model through 3 kinds of stepwise logistic regression analysis.

**Figure 1:**
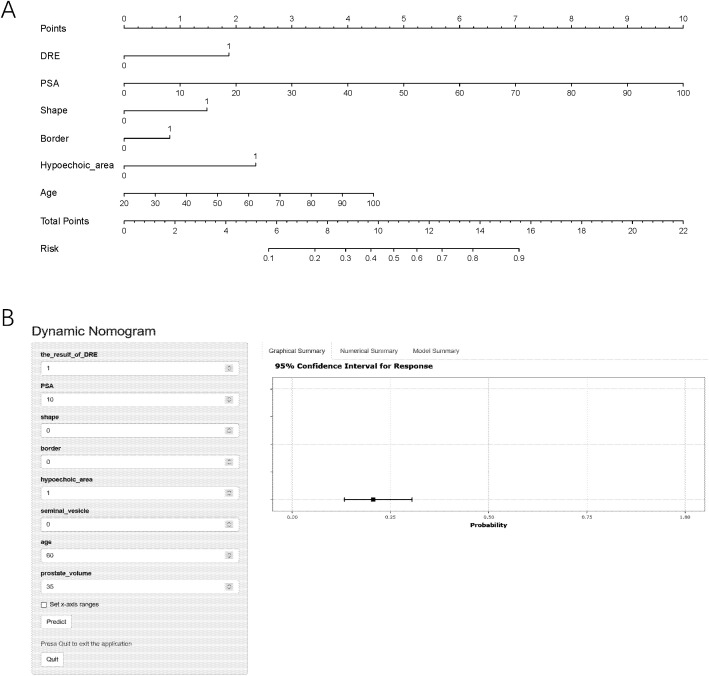
The nomogram prediction model **(A)** and an example of nomogram to predict the risk of prostate cancer via the online calculator **(B)**. PSA, prostate-specific antigen; DRE, digital rectal examination; csPCa, clinically significant prostate cancer. Note: To use the nomogram, please first drew a line from each parameter value to the score axis, added the scores of all parameters, and finally drew a line from the total score axis to determine the probability of csPCa.

The AIC for three models were all 7614.8. We decided to use backward logistic regression to develop the final model. The regression coefficient, standard error, odds ratio (OR), confidence interval (CI), and *p*-values of the variables are summarized in **Table 2A**. notably, the *p* value of “border” was larger than 0.05 (0.129) but considering that its association was verified through multiple tests of the stepwise regression analysis, we recognize its association with the outcome. Based on this model, we used the diagnosis of csPCa as an outcome and described the impact of each variable on the risk of developing csPCa using a nomogram (**Figure 1A**).

**Table 2A:** Coefficients of the prediction model.

Predictors	Coef.	Std.err	OR (95%CI)	*p* value
Hypoechoic area	1.050	0.225	2.857 (1.842-4.460)	<0.001
Shape	0.660	0.252	1.935 (1.175-3.171)	0.009
Border	0.365	0.240	1.440 (0.895-2.299)	0.129
DRE	0.836	0.226	2.307 (1.479-3.592)	<0.001
PSA	0.045	0.004	1.046 (1.038-1.054)	<0.001
Age	0.025	0.012	1.025 (1.002-1.049)	0.033

DRE, digital rectal examination; PSA, prostate-specific antigen; OR, odd ratio; CI, confidence interval.

### 3.3 Validation and utility of the nomogram prediction model

**Figure 2:**
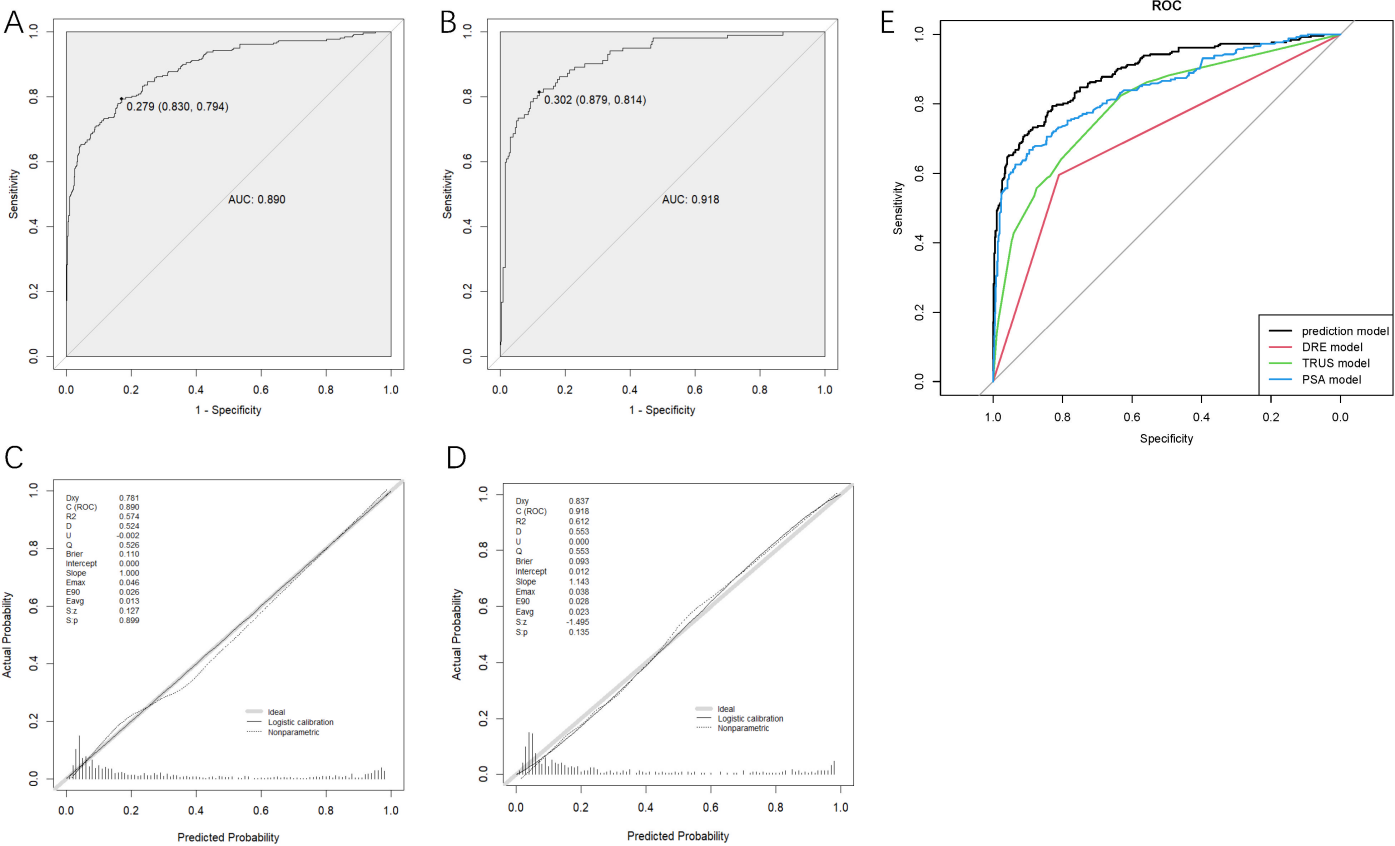
Receiver operating characteristics curves determined by the nomogram model of the training set **(A)** and the validation set **(B)**, the calibration curve of training set **(C)** and validation set **(D)** and receiver operating characteristics curves of four models **(E)**. DRE, digital rectal examination; TRUS, transrectal ultrasound; PSA, prostate-specific antigen.

To verify the predictive ability of the nomogram model for csPCa risk, ROC analysis was performed. **Figures 2A, B** shows that the AUC for the training and validation sets were 0.890 (95%CI: 0.865-0.816) and 0.918 (95%CI: 0.885-0.951), respectively. An AUC of 0.8–0.9 is considered good for this model (19). NPV and PPV are 89.8% and 68.0% respectively. In addition, the optimal critical value in the ROC curve was 0.279 (0.830, 0.794) in the training set and 0.302 (0.879, 0.814) in the validation set. **Table 2B** presents the detailed performance metrics for the two datasets.

**Table 2B:** Performance metrics of the prediction model in training and validation sets.

	Threshold	Specificity	Sensitivity	Accuracy	NPV	PPV
Training set	0.279	0.830	0.794	0.818	0.898	0.680
Validation set	0.279	0.864	0.824	0.852	0.925	0.706

NPV, negative predictive value; PPV, positive predictive value.

The cut-off value of training set (0.279) was assigned into the threshold of validation set.

The degree of calibration of the nomogram models in the modelling and validation sets was assessed using the Hosmer-Lemeshow (H-L) goodness-of-fit test, with a p-value of 0.899 (>0.05) in the training set and 0.135 (>0.05) in the validation set, illustrating no significant difference between the predicted and actual risks. We then visualized the results of the goodness-of-fit test using a calibration curve. **Figures 2C, D** showed that the actual prediction and simulation prediction were similar, indicating good agreement between the nomogram prediction and the actual observation results of csPCa.

**Figure 3:**
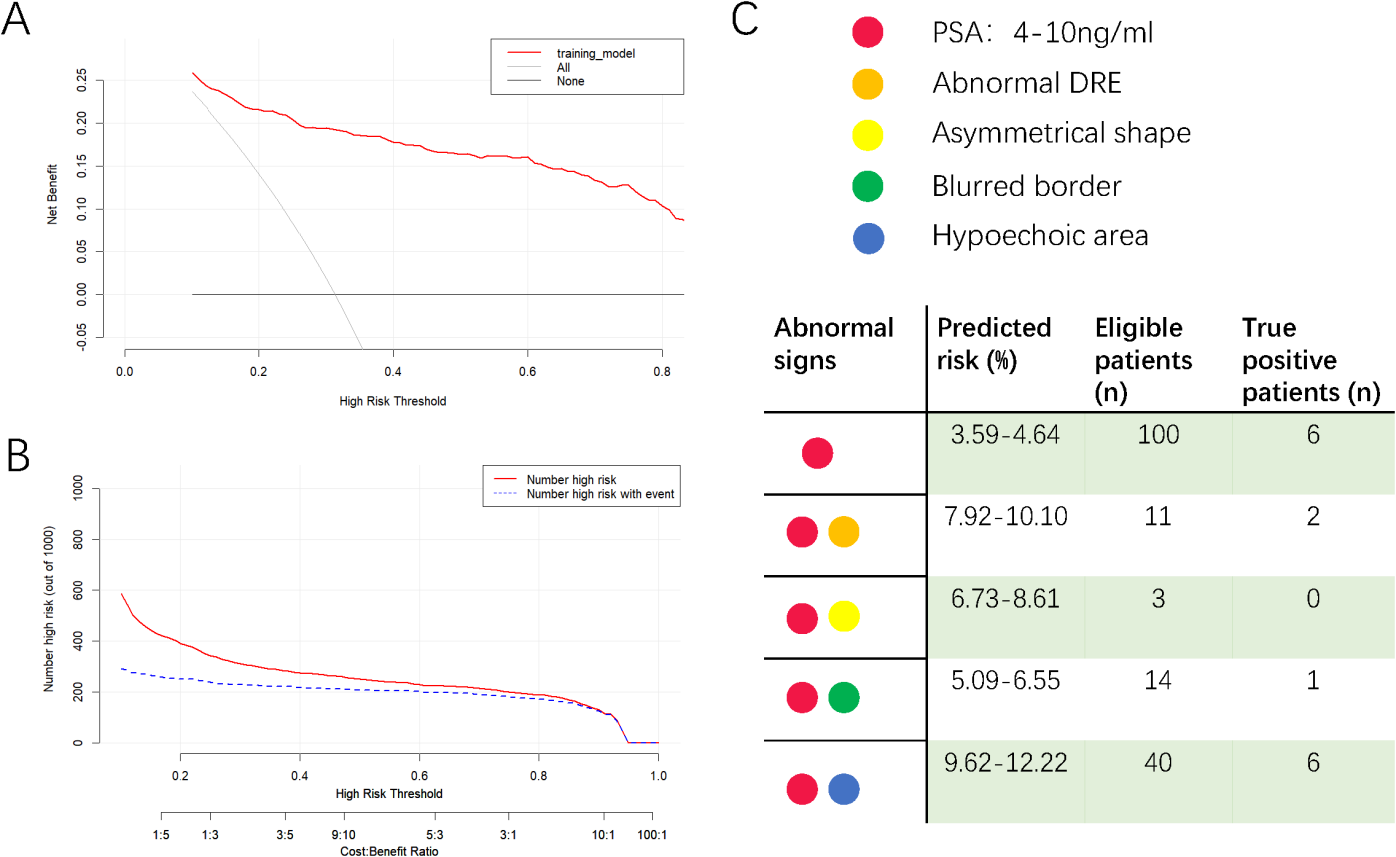
Decision curve analysis **(A)**, clinical impact curve of the model **(B)** and a reference diagram to check the predicted risk **(C)**. In decision curve analysis, X-axis indicates the threshold probability for clinically significant prostate cancer and Y-axis indicates the net benefit of accepting prostate biopsy. In clinical impact curve, the red curve indicates the number of people who are classified as positive; the blue curve represents the number of true positives at each threshold probability. In the reference diagram, clinicians could get the risk of csPCa for patients with only one or two abnormalities. The median age of 71 was applied to calculate the risk.

DCA evaluated the model’s clinical utility (**Figure 3A**). The results indicated that the model provided relatively more net benefits when the threshold probability was greater than 10%. The CIC of the current prediction model is shown in **Figure 3B**. The AUC of the models formed with data from PSA, TRUS, and DRE were 0.844 (95%CI: 0.813-0.875), 0.798 (95%CI: 0.765-0.831), and 0.703 (95%CI: 0.669-0.737), respectively (**Figure 2E**), all smaller than that of the current model with combined data (0.890).

To provide easy access to the proposed model, we developed an online website https://jiwentong0.shinyapps.io/dynnomapp/ to calculate the precise probability of csPCa in patients before prostate biopsy. An example of one patient in our study is demonstrated as an example in **Figure 1B**.

## 4 Discussion

### 4.1 Interpretation of results

This study developed a csPCa risk prediction model based on six predictors (serum PSA level, DRE results, age, prostatic shape, border, and hypoechoic area) recorded using traditional diagnostic methods. The best critical value in the ROC curve was 0.279, and the AUC of the training set was 0.890, indicating a good discriminative ability. The H-L goodness-of-fit test and calibration curve showed good calibration. The DCA and CIC demonstrated good clinical practicality. Data from this study suggest that it would be beneficial for patients with a risk of <27.9% of csPCa to receive active surveillance and dynamic monitoring of PSA. Patients with a risk of >27.9% should opt for prostate MRI or biopsy to obtain a definitive diagnosis.

Univariate and multivariate logistic regression analyses showed that seminal vesicle conditions and prostate volume are irrelevant to csPCa. First, uneven echoes and/or indistinct border of seminal vesicles were defined as abnormal; however, they could have been influenced by benign lesions of the seminal vesicles. A common cause of uneven echoes in ultrasound imaging of seminal vesicles is the presence of stones, which typically appear as well-defined strong echoes with clear boundaries that contrast sharply with normal seminal vesicle tissue. Stones also may be accompanied by acoustic shadowing (20). In contrast, unclear boundaries of the seminal vesicles are often associated with inflammation (21). Park et al. applied bacterial culture to seminal fluid from patients with chronic prostatitis. They determined that approximately 34% of patients exhibited varying degrees of seminal vesicle bacterial infections (21). However, since chronic bacterial prostatitis can also lead to elevated PSA levels, these findings indicate that using only the border clarity of the seminal vesicles is insufficient for differentiating cPCa. Regarding prostate volume, our results were consistent with previous studies about the association between prostate volume and PCa (22). Although BPH and csPCa frequently coexisted among patients in our analysis, volume was not considered an influencing factor for csPCa.

In **Figure 2E**, the AUC of the PSA model was 0.844, better than those of the DRE and TRUS models, highlighting the value of PSA testing. Per convention, the cut-off value of total PSA is 4 μg/mL. However, according to the online calculator, a 71-year-old (median age of the dataset) patient with a PSA of 4 μg/ml and without any other positive index only has a probability of 3.6% to be diagnosed with csPCa. We then attempted to assign the figures of total PSA in the gray zone (4–10 ng/mL) (23) to the calculator and found that the probability increased from 3.6% to 4.6%, which was still lower than the cut-off value of 27.9% in the prediction model. Therefore, for asymptomatic patients with mildly elevated (4–10 ng/ml) PSA levels, our results moderately opposed immediate prostate biopsy and supported short-term monitoring. In order to provide convenience for clinicians to check the risk of csPCa quickly for suspicious patients with PSA within gray zone and accompanying another single abnormality, we designed **Figure 3C** for reference. The number of eligible patients in the dataset and true positive figures were also shown.

### 4.2 Existing csPCa prediction models

We conducted a systematic search of PubMed, Embase, and MEDLINE databases using the subject headings “clinically significant/high-grade prostate cancer,” “risk assessment,” and “model/prediction/score” and were able to identify multiple csPCa prediction models with various predictors. The first two csPCa risk prediction models to be developed were PCa Prevention Trial Risk Calculator 2.0 for high-grade PCa (PCPTRC-HG) and the European Randomized Study of Screening for PCa Risk Calculator for high-grade PCa (ERSPCRC-HG). However, Asian investigators found that racial, environmental, and genetic differences yielded unsatisfactory results when these Western calculators were applied to Asian patients (24). This issue prompted the development of csPCa models tailored specifically to Asian patients, but there were two main concerns. Firstly, the majority of subsequently developed csPCa prediction models were based on MRI results, aligning with the recommendation of MRI for biopsy optimization from the European Association of Urology and American Urological Association (8, 9). However, the necessity of routine pre-biopsy MRI has been challenged. A study suggested that negative MRI results are insufficient to omit biopsy because of relatively low NPV (approximately 85%) (11), and other studies concluded that the diagnostic accuracy and PPV of prostate MRI for csPCa was widely variable (26%−75% and 27%−44% respectively) (10, 25). On the one hand, prostate MRI can increase the upfront diagnostic costs, making patients from developing and underdeveloped areas reluctant to undergo MRI, especially after informing them that a biopsy cannot be omitted even if prostate MRI yields negative results. Hospitals in rural areas or primary care clinics may lack MRI facilities, limiting the applicability of these prediction models. These concerns highlight the importance of developing more cost-effective models, which lead to the second issue. After systematic search, we identified four csPCa models based on inexpensive tests (26–29), such as serum PSA, DRE, TRUS, and post-void residual urinary volume. Only one study used TRUS results as predictor (29), they defined abnormality as hypoechoic area. However, as a basic and simple diagnostic tool for PCa, TRUS could provide not only condition of echo, but also information about shape, border, and seminal vesicle. Existing studies did not incorporate all these diagnostic information into prediction models to improve the clinical value of TRUS.

### 4.3 Strengths and limitations

In response to the above problems, the characteristics and advantages of the current prediction model are in two folds. First, our model was developed based on clinical data of Asian patients, thus making it suitable for application in Asia to prevent unnecessary biopsy, especially in communities and primary hospitals, because the variables used to construct the predictive model are cheap and simple to obtain. Second, our model improved the diagnostic value of three traditional diagnostic methods, particularly included all available diagnostic information about prostate in TRUS.

However, this study had some limitations. First, the model we developed was based on a retrospective study at a single centre. Thus, we were subject to the inherent biases of this type of analysis. Because the majority of patients were Chinese, we could not rule out the possibility that the generalizability would not be applicable to other regions. Finally, the PSA model exhibits a relatively higher AUC (0.844), compared with the AUC of PSA from previous literatures (ranging from 0.660 to 0.799) (30, 31), suggesting a potential selection bias in the patient cohort. This bias might stem from the fact that the majority of patients included in the study come from underdeveloped cities of Northeast China Region, having relatively weak health awareness, especially in the beginning of 2000s. When they seek medical intervention in our centre, they accordingly have higher PSA (median: 17.00 ng/mL) and age (median: 71 years) compared with other studies. In the future, with the gradual improvement of health consciousness, this discrepancy is expected to narrow. We will continue to record new patients and expand our database to improve the accuracy of our model and validate above. explanation.

### 4.4 Future prospects

Due to the difference of environment and race, current model might not provide highest accuracy for patients in other countries. Based on previous comparison studies about Western and Asian models, this flaw might be widespread in the majority of modelling studies. What we truly hope is that our study design could serve as a template to encourage investigators from various continents and countries to develop models specific for their local patients. The extensive usage of these models could play a role of triage test for further MRI or biopsy and significantly reduce upfront cost for patients in developing countries and undeveloped regions. We would provide the guideline of constructing prediction models via R software in **Supplementary Material 3**. Additionally, in the process of literature search, we noticed that other csPCa prediction models applying biomarkers like gene mutations, PCA3 and [-2]proPSA could illustrate good diagnostic power (32–34). With the development of clinical laboratory science and decreasing of the cost of laboratory tests, it is hopeful to incorporate novel biomarkers into current model and get better diagnostic capability.

## 5 Conclusion

In summary, we constructed and validated a csPCa risk prediction model by analysing patients’ clinical data. This model successfully incorporated the results of PSA, DRE, and TRUS and had a good predictive ability. With this prediction model, we aim to provide a cost-effective, non-invasive, and highly accurate risk prediction tool to facilitate early csPCa detection and reduce overdiagnosis in Asia and other underdeveloped areas.

## Data Availability

The raw data supporting the conclusions of this article will be made available by the authors, without undue reservation.

## Supplementary material

Supplementary Material 1 The abnormal examples in transrectal ultrasonography. **(A)**: The borderline of the left part of prostate is unclear compared to the right border. **(B)**: The shape of prostate is asymmetrical. **(C)**: There is a hypoechoic area on the left side of the prostate. **(D)** The seminal vesicle has uneven echoes and indistinct border.
