# Supplementary figures and images for: Construction of a clinically significant prostate cancer risk prediction model based on traditional diagnostic methods
Source: Front Oncol. 2024 Dec 20;14:1474891. doi: 10.3389/fonc.2024.1474891 (PMC11695187; doi:10.3389/fonc.2024.1474891)

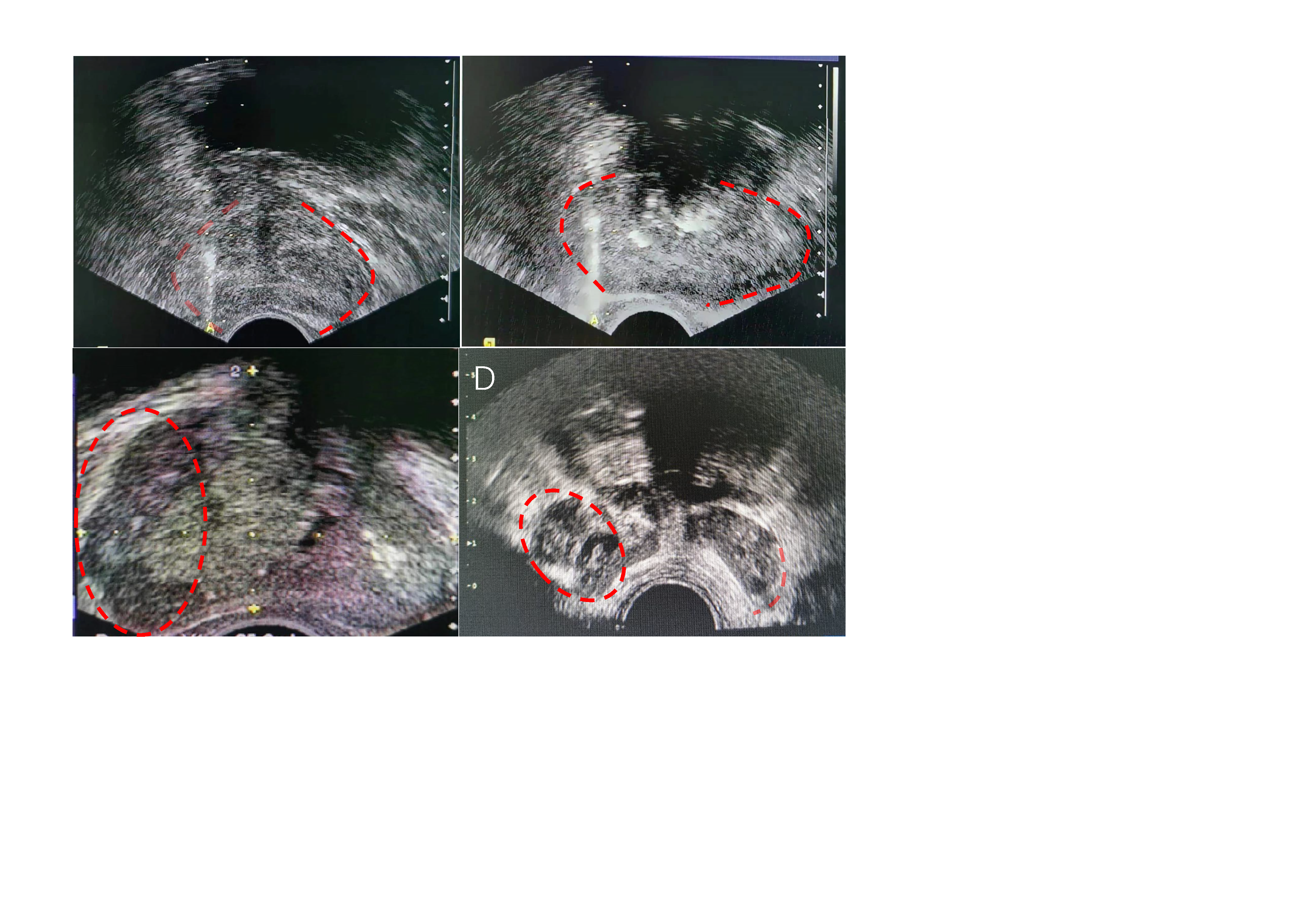

Supplement: Supplementary Material 1 — The abnormal examples in transrectal ultrasonography. (A): The borderline of the left part of prostate is unclear compared to the right border. (B): The shape of prostate is asymmetrical. (C): There is a hypoechoic area on the left side of the prostate. (D) The seminal vesicle has uneven echoes and indistinct border. [file Image1.tif]
