# Supplementary material for: Construction of a clinically significant prostate cancer risk prediction model based on traditional diagnostic methods
Source: Front Oncol. 2024 Dec 20;14:1474891. doi: 10.3389/fonc.2024.1474891 (PMC11695187; doi:10.3389/fonc.2024.1474891)
Supplement: Supplementary file 2 [file DataSheet1.docx]

| Supplementary Material 2A. Comparison of continuous variables before and after multiple imputation | | | |
| --- | --- | --- | --- |
| Variables | Before interpolation | After interpolation | *p* value |
| Age (IQR,year) | 71 (64, 76) | 71 (64, 76) | 0.97 |
| PSA (IQR,ng/ml) | 16.1 (8.3, 35.0) | 17.1 (8.8, 39.7) | 0.25 |
| PV (IQR,cm^3^) | 55.6 (36.4, 87.9) | 55.6 (36.3,87.7) | 0.93 |

| Supplementary Material 2B. Comparison of binary variables before and after multiple imputation | | | | |
| --- | --- | --- | --- | --- |
| Variables | Negative | Positive | X-squared | *p* value |
| **DRE** |  |  | 0.09 | 0.76 |
| Before interpolation | 720 | 342 |  |  |
| After interpolation | 819 | 371 |  |  |
| **Border** |  |  | 0.00 | 1.00 |
| Before interpolation | 762 | 434 |  |  |
| After interpolation | 762 | 434 |  |  |
| **Shape** |  |  | 0.00 | 1.00 |
| Before interpolation | 894 | 302 |  |  |
| After interpolation | 894 | 302 |  |  |
| **Hypoechoic area** |  |  | 0.00 | 1.00 |
| Before interpolation | 650 | 546 |  |  |
| After interpolation | 650 | 546 |  |  |
| **Seminal vesicle** |  |  | 0.26 | 0.61 |
| Before interpolation | 853 | 124 |  |  |
| After interpolation | 1054 | 142 |  |  |
| *PSA, prostate-specific antigen; IQR, interquartile range; DRE, digital rectal examination*  Note: *p*>0.05 indicates that there is no statistically significant difference of data before and after multiple imputation. | | | | |
