# Supplementary material for: Construction of a clinically significant prostate cancer risk prediction model based on traditional diagnostic methods
Source: Front Oncol. 2024 Dec 20;14:1474891. doi: 10.3389/fonc.2024.1474891 (PMC11695187; doi:10.3389/fonc.2024.1474891)
Supplement: Supplementary file 3 [file DataSheet2.docx]

**Step 1.1 conduct multiple imputations**

install.packages("VIM")

library(VIM)

install.packages("mice")

library(mice)

df<-mice(result0,m=4,method="cart",seed=1024)

result1<-complete(df,action=4)# multiple imputations

aggr(data)#view the result of interpolation

**Step 1.2 check the differences before and after** **multiple imputations**

result_man<-wilcox.test(result0$PSA,result1$PSA)# Mann-Whitney U test for continuous variables

x2data1<-rbind(x2test[1,],x2test[2,])

x2chisq<-chisq.test(x2data1)#chi-square test for categorical variables

**Step 2.1 Divide dataset into training and validation sets**

set.seed(1234)

training_index<-sample(x=1:1196,size=1196*0.7,replace=F)

validation_index<-c<-c(1:1196)[-training_index]

training_set<-result1[training_index,]

validation_set<-result1[validation_index,]#70%for training 30%for validation

**Step 2.2 check the differences between validation and training sets**

t.test(training_set$SV,validation_set$SV,var.equal = TRUE)

table(result1$SV)

table_var<-table(result1$SV)

prop_var<-prop.table(table_var)

prop_var#for categorical variables

t.test(training_set$PSA,validation_set$PSA,var.equal = TRUE)#for continuous variables

**Step 3.1 Univariate logistic regression analysis was performed on the training set to screen for potential factors preliminarily**

logit<-function(x,y,z){

require(broom)

require(tableone)

xx<-c(NA,NA,NA,NA,NA,NA,NA)

xx<-matrix(xx,1,7)

table<-data.frame(xx)

names(table)<-c("term","estimate","std.error","statistic",

"p.value","exp(doef)[confint]","p")

table<-table[-1,]

for(i in 1:length(x)){

glm.log<-glm(substitute(y~x,list(x=as.name(x[i]),y=as.name(y))),

family=binomial(link="logit"),data=z)

table11<-ShowRegTable(glm.log,exp=TRUE,digits=3,pDigits = 3,

printToggle=TRUE,quote = FALSE,ciFun = confint)

table12<-tidy(glm.log)

table2<-cbind(table12,table11)

table<-rbind(table2,table)

table<-table[-1,]

}

return(table)

}

x<-c("age","PSA","DRE","PV","border","shape","hypo","SV")

y<-c("PD")

z<-training_set

xx<-logit(x,y,z)#hypo,hypoechoic area; SV, seminal vesicle; PV, prostate volume; PD, pathological diagnosis

sign.x<-xx$term[xx$p.value<0.05]

(form<-as.formula(paste0('PD~',paste0(sign.x,collapse='+')))) #extract predictors with p<0.05

**Step 3.2 Further screen potential factors based on multivariate regression analysis and develop the final model**

multi.model<-glm(form,data=training_set,family=binomial)# multivariate regression analysis

model.backward<-step(multi.model,direction="backward",trace=0)

summary(model.backward)#backward stepwise logistic regression

modelX<-glm(PD~1,data=training_set,family=binomial)

model.forward<-step(modelX,scope=list(upper=~hypo+ shape + border + DRE + PSA + age,

lower=~1),data=training_set,family=binomial,direction="forward",

trace=0)

summary(model.forward)#forward stepwise logistic regression

model.both<-step(multi.model,direction="both",trace=0)

summary(model.both)# forward-backward stepwise logistic regression

**Step 3.3 calculate AIC of four models and choose the model with the lowest AIC**

AIC(multi.model,model.forward,model.backward,model.both)

exp(cbind("OR"=coef(model.backward),confint(model.backward)))#view OR

**Step 3.4 develop nomogram**

library(writexl)

write_xlsx(training_set,path="training_set1")#download the dataset

require(rms)

ddist<-datadist(training_set1)

options(datadist='ddist')#package the data

model.step2<-lrm(Diagnosis_of_csPCa ~ DRE + PSA + Shape + Border + Hypoechoic_area + Age,data=training_set1,x=TRUE,y=TRUE)#use standard names for variables

nomo<-nomogram(model.step2,

maxscale = 10,

lp=T,

fun=function(x)1/(1+exp(-x)),

fun.at=seq(0,1,by=0.1),

funlabel="Risk")

plot(nomo,

lplabel="Linear Predictor",

points.label = 'Points',

xfrac=.15,

total.points.label = 'Total Points')#nomogram

**Step 4.1 validation**

model.backward<-glm(PD ~ age + hypo + shape + border + DRE + PSA,

data=training_set,

family=binomial(logit))

training_set$pred.back<-predict(model.backward,

newdata=training_set,

type="response")

validation_set$pred.back<-predict(model.backward,

newdata=validation_set,

type="response")#calculate the predicted risk in training and validation set separately

library(pROC)#load package

roc.training.backward<-roc(training_set$PD,training_set$pred.back)

auc(roc.training.backward)

ci(auc(roc.training.backward))

plot(roc.training.backward,

legacy.axes=TRUE,

print.auc=TRUE,

auc.polygon=FALSE,

lwd=1,

max.auc.polygon=TRUE,

percent=FALSE,

print.thres=TRUE)# ROC curve drawing for training set

roc.validation.backward<-roc(validation_set$PD,validation_set$pred.back)

auc(roc.validation.backward)

ci(auc(roc.validation.backward))

plot(roc.validation.backward,

legacy.axes=TRUE,

print.auc=TRUE,

auc.polygon=FALSE,

lwd=1,

max.auc.polygon=TRUE,

print.thres=TRUE)#ROC curve drawing for validation set

ROC.results<-coords(roc.training.backward,"best",ret="all",transpose=FALSE)

as.matrix(ROC.results)#view parameters of diagnostic performance of training set

ROC.results<-coords(roc.validation.backward,please copy the threshold of training set here,ret="all",transpose=FALSE)

as.matrix(ROC.results) #view parameters of diagnostic performance of validation set

**Step 4.2 Calibration analysis**

library(rms)#load package

val.prob(training_set$pred.back,training_set$PD)

val.prob(validation_set$pred.back,validation_set$PD)# p>0.05 illustrates no significant difference between the predicted and actual risks

library(ResourceSelection)#load package

hoslem.test(training_set$PD,training_set$pred.back,g=10)

hoslem.test(validation_set$PD,validation_set$pred.back,g=10)# Hosmer and Lemeshow goodness of fit

**Step 4.3 DCA curve**

library(rmda)#load package

dca.training_set<-decision_curve(PD~pred.back,

data=training_set,

family=binomial(logit),

thresholds=seq(0.1,by=0.01),

confidence.intervals = 0.95,

study.design = 'cohort')

plot_decision_curve(dca.training_set,curve.names = c('training_model'),

xlim=c(0,0.8),

cost.benefit.axis = FALSE,

col=c('red'),

confidence.intervals = FALSE,

standardize = FALSE)#training set

**Step 4.4 CIC**

plot_clinical_impact(dca.training_set, population.size = 1000,cost.benefit.axis = T,

n.cost.benefits = 8,col = c('red','blue'),

confidence.intervals = F)

**Step 4.5 develop an online calculator**

library(DynNom)#load package

install.packages("rsconnect")

library(rsconnect)

DNbuilder(model.backward1,

covariate="numeric")#make the values in to slider

library(rsconnect)

rsconnect::setAccountInfo(name='name of the account',

token='token of the account',

secret='code of the account')#run the account

accounts(server=NULL)

dir<-getwd()

path<-paste0(dir,"/DynNomapp",collapse="")#set the running path

path# show the running path

rsconnect::deployApp("storage location")
